# Interleukin-17A inhibitors for guttate psoriasis treatment: A retrospective cohort study

**DOI:** 10.1016/j.jdin.2026.01.006

**Published:** 2026-01-31

**Authors:** Xiao-yong Man, Gao-yuan Zhao, Li-ran Ye

**Affiliations:** Department of Dermatology, Second Affiliated Hospital, Zhejiang University School of Medicine, Hangzhou, China

**Keywords:** biologics, clinical practice, guttate psoriasis, IL-17A inhibitor, ixekizumab, secukinumab

*To the Editor:* Guttate psoriasis (GP) is a variant of psoriasis characterized by numerous, small, widely scattered scaly papules and plaques.^1^ Though it is usually self-limiting, most patients require treatment, with some resistant to conventional therapy or even progressing to other types of psoriasis.^2^ Previous reports demonstrated that patients with refractory GP who had failed conventional therapies showed clinical improvement after biologic treatments, such as ustekinumab^3^ and risankizumab.^4^ However, evidence on the effectiveness of interleukin (IL)-17A inhibitors remains limited. This study retrospectively analyzed clinical data of GP patients treated with secukinumab or ixekizumab in a real-world setting to evaluate the effect and safety of IL-17A Inhibitors for GP.

We enrolled 29 GP patients treated with IL-17A inhibitors (Table I). Patients had failed to respond to previous treatment regimens and then received secukinumab (*n* = 13) or ixekizumab (*n* = 16). Disease severity was assessed by psoriasis area and severity index (PASI) scores extracted directly from the medical records. PASI scores at weeks 0, 4, 8, and 12 were recorded among the patients. The inclusion criteria and treatment regimen are described in Supplementary Material 1 (available via Mendeley at https://data.mendeley.com/datasets/np2tdz5wvj/1).

**Table I:** Demographic and clinical characteristics of all patients

Characteristics	Total	Secukinumab	Ixekizumab	*P* value
Patients, no. (%)	29 (100.0)	13 (44.8)	16 (55.2)	
Age, mean ± SD, y	24.76 ± 11.64	19.85 ± 12.52	28.75 ± 9.48	.050
Sex, no. (%)				.066
Male	14 (48.3)	9 (69.2)	5 (31.3)	
Female	15 (51.7)	4 (30.8)	11 (68.7)	
BMI, mean ± SD, kg/m^2^	21.14 ± 4.23	21.28 ± 4.86	21.03 ± 3.81	.948
Psoriasis duration, median (IQR), y	2.0 (7.9)	1.0 (15.4)	2.0 (7.6)	.846
Family history, no. (%)	3 (10.3)	2 (15.4)	1 (6.3)	.537
History of previous URTI, no. (%)	14 (48.3)	5 (38.5)	9 (56.3)	.462
Elevation of ASO titers, no. (%)	10 (34.5)	3 (23.1)	7 (43.8)	.433
Baseline PASI, mean ± SD	14.52 ± 8.23	14.19 ± 9.37	14.78 ± 7.49	.826
Baseline BSA%, mean ± SD	25.50 ± 18.54	29.08 ± 22.96	22.59 ± 14.13	.650
Previous treatment, no. (%)	29 (100.0)	13 (100.0)	16 (100.0)	
Topical drugs∗, no. (%)	21 (72.4)	10 (76.9)	11 (68.8)	.697
Phototherapy, no. (%)	2 (6.9)	0 (0.0)	2† (12.5)	.488
Antibiotics‡, no. (%)	17 (58.6)	8 (6.2)	9 (56.3)	1.000
Acitretin, no. (%)	1 (3.4)	1 (7.7)	0 (0.0)	.488
Chinese herb, no. (%)	5 (17.2)	3 (23.1)	2 (12.5)	.632
Biologics, no. (%)	1 (3.4)	1§ (7.7)	0 (0.0)	.488

*ASO*, Anti-streptolysin O; *BMI*, body mass index; *BSA*, body surface area; *IQR*, interquartile range; *PASI*, psoriasis area and severity index; *SD*, standard deviation; *URTI*, upper respiratory tract infection.

∗ Topical drugs included topical corticosteroids, vitamin D derivatives, or tacrolimus.

† Both were treated with narrow-band ultraviolet B (NB-UVB).

‡ Antibiotics included intramuscular benzathine benzylpenicillin, oral azithromycin, or oral cephalosporin.

§ One patient had previously received adalimumab for 2 years but discontinued it for 4 months owing to loss of efficacy.

At week 4, 23 of 29 patients (79.3%) reached PASI75, 65.5% reached PASI90, and 51.7% reached PASI100. By week 12, almost all patients achieved PASI75 (96.6%) and PASI90 (96.6%), and 86.2% achieved PASI100. Specifically, at week 12, the mean PASI score decreased from 14.52 ± 8.23 to 0.16 ± 0.51 (*P* < .05) after IL-17A inhibitors. In both secukinumab and ixekizumab groups, mean PASI scores gradually decreased from baseline to week 12 (Fig 1). Ixekizumab seemed to exhibit an earlier response onset, which was demonstrated by lower PASI values at week 4 compared with secukinumab (Fig 1).

**Fig 1:**
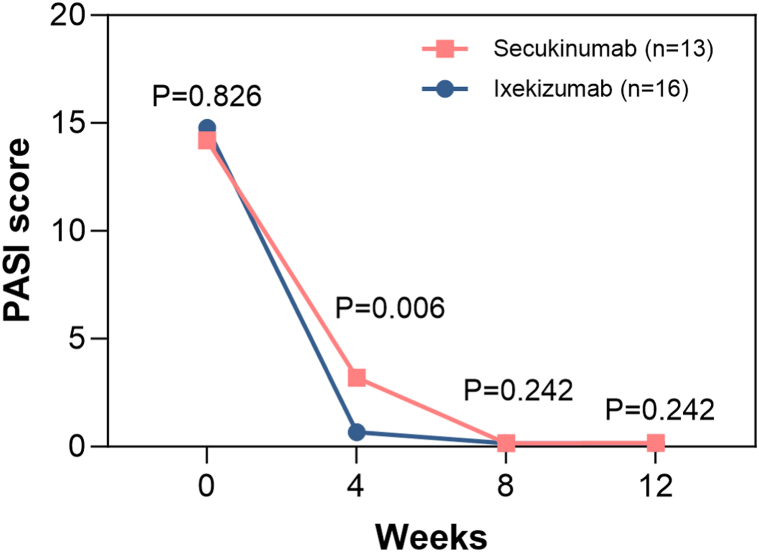
Results within 12 weeks: PASI scores of patients treated with secukinumab or ixekizumab. *PASI*, Psoriasis area and severity index.

After week 12, part of patients discontinued or extended of dosing intervals (see Supplementary Material 2, available via Mendeley at https://data.mendeley.com/datasets/np2tdz5wvj/1). Five patients discontinued secukinumab and 7 discontinued ixekizumab after 12 weeks because of clearance, and 4 (80.0%) maintained PASI75 up to 70 weeks off secukinumab, and 5 (71.4%) maintained PASI75 up to 50 weeks off ixekizumab, similar to previous cases indicating a durable control of disease.^5^ To alleviate the financial burden, 3 of 6 (50%) patients under secukinumab, and 4 of 7 (60%) patients under ixekizumab effectively extended their dosing interval without experiencing a relapse during the observation period. Interestingly, there was 1 patient in each group to undergo biologics switch due to loss of PASI50, and the use of another IL-17A inhibitor also led to PASI75 again, confirming the efficacy of IL-17A inhibitors. At follow-up, patients experiencing flare could achieve PASI75 response again by reinjection, continuing the standard maintenance regimen, or switching to another IL-17A inhibitor.

For safety, after secukinumab treatment, 1 patient presented with conjunctivitis, and another presented with hordeolum and cutaneous infection. After ixekizumab treatment, no adverse events were observed.

In conclusion, both secukinumab and ixekizumab exhibited favorable responses and were well-tolerated in GP, inducing a rapid clinical response and leading to durable disease control. However, larger prospective studies are warranted to validate these observations.

## Conflicts of interest

None disclosed.
